# Molecular detection of human *Plasmodium* species using a multiplex real time PCR

**DOI:** 10.1038/s41598-023-38621-9

**Published:** 2023-07-14

**Authors:** Yassamine Lazrek, Célia Florimond, Béatrice Volney, Manon Discours, Emilie Mosnier, Sandrine Houzé, Stéphane Pelleau, Lise Musset

**Affiliations:** 1 Laboratoire de Parasitologie, World Health Organization Collaborating Center for Surveillance of Antimalarial Drug Resistance, Centre Nationale de Référence du Paludisme, Institut Pasteur de la Guyane, Cayenne, French Guiana; 2 Aix Marseille Univ, INSERM, IRD, SESSTIM, Sciences Economiques & Sociales de la Santé & Traitement de l’Information Médicale, Aix Marseille Institute of Public Health ISSPAM, 13385 Marseille, France; 3 IRD, MERIT, Université Paris Cité, 75006 Paris, France; 4 Centre National de Référence du Paludisme, AP-HP, Hôpital Bichat-Claude Bernard, 75018 Paris, France; 5 Infectious Disease Epidemiology and Analytics Unit, Institut Pasteur, Université Paris Cité, Paris, France

**Keywords:** Microbiology, Molecular biology

## Abstract

Molecular detection methods have revealed higher sensitivity and specificity than conventional microscopy or rapid diagnostic tests for malaria diagnosis. In this study, we implemented, evaluated and validated according to the ISO 15,189 requirements, a multiplex real-time PCR assay to detect and identify the five human malaria parasites. DNA samples were extracted from whole blood or dried blood spots drawn from patients. Based on the External Quality Assessment (whole blood), this method shows 100% sensitivity and specificity. This PCR detected *P. vivax* up to 0.25 p/µl, *P. falciparum* and *P. knowlesi* up to 0.5 p/µl, *P. ovale* up to 1 p/µl and *P. malariae* up to 5 p/µl of blood. From blood spots (extraction from four punches), it detected *P. vivax* at 5 p/µl, *P. falciparum*, *P. ovale* and *P. knowlesi* at 20 p/µl and *P. malariae* at 125 p/µl. In conclusion, this quantitative PCR shows excellent performance, is easy to use and DNA saver. It is especially useful to actively screen large population groups and identify the five human malaria parasites in a context of low malaria transmission.

## Introduction

Malaria is one of the deadliest diseases and has already claim millions of life throughout the world, 619,000 in 2021^1^. This vector-borne disease is caused in Humans by five different species of *Plasmodium* genus*, P. falciparum*, *P. vivax*, *P. malariae*, *P. ovale*, and *P. knowlesi*^2^. The last one has been recently transmitted to Humans from primates in Malaysia^3^. *P. falciparum* generates the highest mortality and morbidity rate. However, *P. vivax* is the most widely distributed across all endemic areas and the second largest contributor to clinical malaria worldwide^1^. This species was originally considered benign but is now recognized as a cause of serious morbidity and mortality^4,5^. Accurate malaria diagnosis is crucial to reduce presumptive treatment. Microscopy is the main tool to diagnose malaria together with rapid diagnosis tests (RDTs). Their performances allow the diagnosis of patient with a parasitemia up to 10 parasites/µl of blood. Owing to their sensitivities, they are limited to identify asymptomatic carriers presenting low parasitemia. However, addressing these parasite reservoirs is crucial for national malaria programs moving forward malaria elimination. These programs call for novel diagnosis methods, highly sensitive but costly effective to actively screen large population groups^6^.

The molecular amplification of the small 18S subunit of ribosomal RNA (18S rRNA), first implemented by Snounou et al. using a nested PCR technique, is the most widely used molecular diagnostic tools in medical laboratories or in research programs^7,8^. The *18S rRNA* gene is commonly the target because of its 5 to 7 copies per genome, which increases sensitivity^9^. It is also conserved in all *Plasmodium* species with specific parts for each of them.

Since then, molecular techniques have evolved and now include, real-time PCR, Reverse Transcriptase PCR, Nucleic Acid Sequence-Based Amplification (NASBA) and LAMP (Loop-mediated isothermal amplification). These PCR can detect a wide range of gene targets and are 10 to 100 times more sensitive than microscopy^10,11^. Compare to the original nested-PCR, they also give results more rapidly (within an hour compared to 4.5–10 h).

In this study, we developed and evaluated a sensitive and specific multiplex real-time PCR procedure to detect and identify the five malaria parasites transmitted to Humans (*P. falciparum*, *P. vivax, P. malaria*, *P. ovale* and *P. knowlesi*). Adapted from Shokoples et al.^12^ and de Canale et al.^13^, two real-time multiplex PCR have been implemented, one targeting *P. falciparum* and *P. vivax* and another targeting *P. malariae*, *P. ovale* and *P. knowlesi*. The multiplex approach has been chosen in order to minimize DNA, time and money consumption. DNA extraction was standardized and controlled using a third simplex PCR targeting the human macroglobulin gene. If a quick and large screening is required during active case detection, we also implemented a *Plasmodium spp.* PCR method adapted from Hassanpour et al.^14^. Those methods have been accredited according to the ISO 15,189 medical biology norm to analyze DNA extracted from venous or capillary blood.

## Results

### Repeatability, reproducibility and internal quality controls of 18S screening real time PCR

The coefficients of variation (CVs) for intra-assay repeatability and inter-assay reproducibility were showed in Tables 1 and 2.

**Table 1:** Repeatability of the real-time PCR approach to detect *Plasmodium* species.

	*P. falciparum*	*P. vivax*	*P. malariae*	*P. ovale*	*P. knowlesi*
Coefficient of variation at high-parasitemia*	0.3%	2.4%	1.0%	0.5%	0.6%
Coefficient of variation at low-parasitemia°	0.6%	3.1%	0.5%	2.2%	1.0%
Number of replicates (n)	n = 3	n = 3	n = 3	n = 3	n = 3

Detailed values for high (*) and low (°) parasitemia samples are listed in Table 9.

**Table 2:** Reproducibility of the real-time PCR approach to detect *Plasmodium* species.

	*P. falciparum*	*P. vivax*	*P. malariae*	*P. ovale*	*P. knowlesi*
Internal control (copy/µl)	3200	3300	100	3100	1000
Coefficient of variation	1.2%	1.0%	3.6%	2.5%	2.5%
Number of replicates (n)	n = 78	n = 80	n = 90	n = 95	n = 58

Internal quality controls (IQC) are included in each sample series. They were first analyzed according to the Levy-Jennings rules based on the coefficient of variation established with repeatability (Table 1). After the first months of implementation, reproducibility values have been determined (Table 2), then simplify and harmonized at 2.5% for all species.

**Figure 1:**
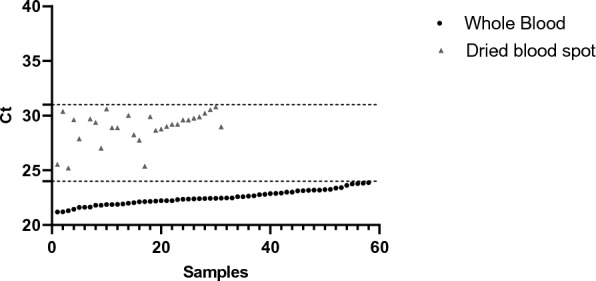
Human β2Megaglobuline Ct values.

To validate the DNA extraction, a threshold was defined for the internal control human β2Megaglobuline. Based on the results obtained on 58 samples of the “Palustop project”^15^ from whole blood (WB) and 31 from dried blood spot (DBS) of the “NRC collection”, Ct ranged between 21 and 24 (mean 22.5 ± 0.6) and between 25.2 and 32.3 (29.1 ± 1.7, Fig. 1) for WB and DBS, respectively. Therefore, the thresholds to validate DNA extraction were fixed, Ct ≤ 24 for WB DNA and, Ct ≤ 33 for DBS DNA.

### Analytical sensitivity and specificity

The performance of this species-specific real-time PCR was compared to our External Quality Assessment (EQA) to validate the ability of the method to diagnose malaria during patient care. This included samples collected in tubes or dried blood spots infected by one of the five species at different parasitemia, from 0.018 parasites per µl (p/µl) up to 1.1 × 10^6^ p/µl. We also tested several negative EQA. The sensitivity and specificity of the technique for all species in whole blood were 100% (Table 3).

**Table 3:** Sensitivity and specificity regarding the external quality assessment on 200 µl of blood.

		Methods	Positive EQA	Negative EQA	Sensitivity (%)	Specificity (%)	PPV (%)	NPV (%)
*P. falciparum*	*Real time PCR*	Positive	22	0	100 ± 13.6	100 ± 15.8	100 ± 13.6	100 ± 15.8
		Negative	0	19				
		Total	22	19				
*P. vivax*		Positive	16	0	100 ± 18.8	100 ± 15.8	100 ± 18.8	100 ± 15.8
		Negative	0	19				
		Total	16	19				
*P. malariae*		Positive	10	0	100 ± 30.0	100 ± 15.8	100 ± 30.0	100 ± 15.8
		Negative	0	19				
		Total	10	19				
*P. ovale*		Positive	9	0	100 ± 33.3	100 ± 15.8	100 ± 33.3	100 ± 15.8
		Negative	0	19				
		Total	9	19				
*P. knowlesi*		Positive	9	0	100 ± 33.3	100 ± 15.8	100 ± 33.3	100 ± 15.8
		Negative	0	19				
		Total	9	19				

*EQA* External Quality Assessment, *NPV* negative predictive value, *PPV* positive predictive value. Parasitemia of the positive samples: *P. falciparum* from 0.1 to 1.10^6^ p/µl, *P. vivax* from 0.018 to 2.10^5^ p/µl, *P. malariae* from 1 to 125 p/µl, *P. ovale* from 1 to 200 p/µl and *P. knowlesi* from 1 to 1.10^4^ p/µl.

For DNA extracted on dried blood spot (Table 4), the sensitivity was 100% for *P. vivax, P. ovale* and *P. knowlesi*. However, for *P. falciparum* and *P. malariae*, the sensitivity and the negative predictive value (NPV) were 73% and 100%, 43% and 100%, respectively. The specificities were at 100%*.*

**Table 4:** Sensitivity and specificity regarding the External quality assessment in dried blood spot.

		Methods	Positive EQA	Negative EQA	Sensitivity (%)	Specificity (%)	PPV (%)	NPV (%)
*P. falciparum*	*Real time PCR*	Positive	8	0	73 ± 26	100 ± 25	73 ± 26	100 ± 25
		Negative	3	12				
		Total	11	12				
*P. vivax*		Positive	6	0	100 ± 50	100 ± 25	100 ± 50	100 ± 25
		Negative	0	12				
		Total	6	12				
*P. malariae*		Positive	3	0	43 ± 37	100 ± 25	43 ± 37	100 ± 25
		Negative	4	12				
		Total	7	12				
*P. ovale*		Positive	4	0	100 ± 75	100 ± 25	100 ± 75	100 ± 25
		Negative	0	12				
		Total	4	12				
*P. knowlesi*		Positive	3	0	100 ± 100	100 ± 25	100 ± 100	100 ± 25
		Negative	0	12				
		Total	3	12				

*EQA* External Quality Assessment, *NPV* negative predictive value, *PPV* positive predictive value. Parasitemia of the positive samples: *P. falciparum* from 0.2 to 1.1 10^6^ p/µl, *P. vivax* from 5 to 400 p/µl, *P. malariae* from 2.3 to 800 p/µl, *P. ovale* from 20 to 280 p/µl and *P. knowlesi* from 2 to 20 p/µl.

### Real-time PCR limits of detection (LoD)

In diagnosis, when the parameter is close to the limit of detection, a sample analyzed several times could be either positive or negative. When a result is based on quantitative data, it is crucial to determine the limits of this zone, named the grey zone. Outside this zone, the samples should be 100% positive among replicates while within the grey zone, a percentage could be applied. For these malaria PCRs, the grey zone included Ct > 36. To increase sensitivities, samples were analyzed is triplicate. Within the grey zone and according to the specificities of the method, as soon as a sample was positive among the triplicate, the result was positive for malaria infection.

Based on these rules, the detection limits of these real-time PCR have been compared to the historical nested PCR. After DNA extraction of 200 µl of venous blood, the method detected *P. vivax* up to 0.25 p/µl*, P. falciparum* and *P. knowlesi* up to 0.5 p/µl, *P. ovale* up to 1 p/µl (Table 5) and *P. malariae* up to 5 p/µl (Ct = 39.4 ± 1.5, positive wells 3/3). If we include the grey zone results, this detection limits increase up to 0.06 p/µl for *P. knowlesi*, 0.12 p/µl for *P. falciparum* and *P. vivax* and 1 p/µl for *P. malariae*. When DNA was extracted from capillary blood and collected on filter papers, the method allowed the detection of *P. vivax* infection up to 10 p/µl and *P. falciparum* up to 5 p/µl (Supplementary Table S1).

**Table 5:** Comparison of the detection limits of nested and real time PCR for *P. falciparum*, *P. vivax P. malariae*, *P. ovale* and *P. knowlesi* based on DNA extracted from 200 µl of whole blood.

Parasitemia	(p/µl)	100	20	10	1	0.5	0.25	0.12	0.06	0.03
*P. falciparum*	Nested PCR	+	+	+	+	+	+	*−*	−	−
	Real Time PCR	28.6 ± 0.4	32.1 ± 0.3	32.3 ± 0.1	36.0 ± 0.2	**37.3 ± 0.8**	*36.2* ± *0.7*	*38.0* ± *NA*	−	−
	Positive wells*	3/3	3/3	3/3	3/3	**3/3**	*2/3*	*1/3*	0/3	0/3
*P. vivax*	Nested PCR	+	+	+	+	+	+	*−*	−	−
	Real Time PCR	28.8 ± 0.0	31.0 ± 0.1	31.6 ± 0.1	36.0 ± 0.9	36.6 ± 1.0	**37.6 ± 1.3**	*38.0* ± *NA*	−	−
	Positive wells*	3/3	3/3	3/3	3/3	3/3	**3/3**	*1/3*	0/3	0/3
*P. malariae*	Nested PCR	+	+	+	*−*	−	−	−	−	−
	Real Time PCR	35.4 ± 0.4	38.5 ± 1.0	**38.1 ± 1.0**	*41.0* ± *NA*	−	−	−	−	−
	Positive wells*	3/3	3/3	**3/3**	*1/3*	0/3	0/3	0/3	0/3	0/3
*P. ovale*	Nested PCR	+	+	+	+	−	−	−	−	−
	Real Time PCR	31.0 ± 0.2	33.0 ± 0.2	35.1 ± 0.8	**36.3 ± 1.2**	−	−	−	−	−
	Positive wells*	3/3	3/3	3/3	**3/3**	0/3	0/3	0/3	0/3	0/3
*P. knowlesi*	Nested PCR	+	+	+	−	−	*−*	*−*	*−*	−
	Real Time PCR	29.0 ± 0.2	31.4 ± 0.2	34.0 ± 0.2	37.6 ± 0.3	**37.3 ± 0.5**	*38.0* ± *0.5*	*38.6* ± *NA*	*40.3* ± *NA*	−
	Positive wells*	3/3	3/3	3/3	3/3	**3/3**	*2/3*	*1/3*	*1/3*	0/3

Results are presented in mean Ct (cycle threshold) ± standard deviation according to the level of parasitemia (p/µl, parasite/µl). In bold and italics, the LoD chosen. *NA* non-applicable. *Number of positive well in each real time triplicate.

The real time PCR targeting the genus *Plasmodium spp.* demonstrated similar limits of detection than the PCR-species specific (Table 6).

**Table 6:** Determination of the limits of detection for *Plasmodium *spp*.* real time PCR targeting 18S genes in whole blood.

Parasitemia (p/µl)		100	20	10	1	0.5	0.25	0.12	0.06	0.03	0.015
*Plasmodium *spp.	*P. falciparum*	26.4 ± 0.1	29.0 ± 0.3	30.0 ± 0.3	**33.0 ± 0.7**	*34.0* ± *0.7*	*36.2* ± *1.2*	*37.2* ± *NA*	*39.0* ± *NA*	−	−
	*Positive wells*	3/3	3/3	3/3	**5/5**	*4/5*	*3/5*	*1/5*	*1/5*		
	*P. vivax*	25.5 ± 0.1	28.5 ± 0.2	29.0 ± 0.2	**33.0 ± 0.5**	*34.4* ± *0.5*	*35.0* ± *0.5*	*36.3* ± *NA*	*36.0* ± *0.1*	*36.5* ± *NA*	−
	*Positive wells*	3/3	3/3	5/5	**5/5**	*4/5*	*3/5*	*1/5*	*3/5*	*1/5*	
	*P. malariae*	32.3 ± 0.3	34.6 ± 0.4	**34.2 ± 0.6**	*35.1* ± *0.8*	−	−	−	−	−	−
	*Positive wells*	3/3	3/5	**5/5**	*2/5*						
	*P. ovale*	28.0 ± 0.3	30.0 ± 0.2	31.4 ± 0.4	**34.2 ± 1.2**	*36.2* ± *0.2*	−	−	−	−	−
	*Positive wells*	3/3	5/5	3/3	**5/5**	*2/4*					
	*P. knowlesi*	25.6 ± 0.1	27.6 ± 0.1	30.3 ± 0.1	33.6 ± 0.5	**34.5 ± 1.0**	*35.0* ± *NA*	*35.5* ± *0.1*	−	−	−
	*Positive wells*	3/3	3/3	2/2	2/2	**5/5**	*1/5*	*3/5*			

Results are presented in mean Ct (cycle threshold) ± standard deviation according to the level of parasitemia (p/µl, parasite/µl). In bold and italics, the LoD chosen. *NA* non-applicable. *Number of positive well in each real time triplicate.

### Detection of mixed infections

Cross-reactions between species have been assessed in simplex amplification for each of the five-malaria species. No cross-reaction has been observed.

The impact of a multiplex strategy on PCR efficiency has been done for *P. vivax* amplification (Supplementary Fig. S1). Ct differences observed for each of the dilution points were between − 0.6% and + 1%. These variations were included within the coefficient of variation of the method. Therefore, the results were not impacted by the use of a combination of two or three primer pairs during the multiplex strategy.

The interferences between species have been assessed using artificially prepared mixed-infections from patient infected blood (Table 7) or using plasmids containing *P. falciparum* and *P. vivax* target sequence (Supplementary Table S2). Results from the two types of matrix showed an impact on the Ct values when one of the species was predominant. The identification of the minor one was therefore more difficult. For example, a *P. vivax* infection in minority among *P. falciparum* parasites could not be detected when the major species generated a Ct greater than 29 (Table 7). Based on our experience, in case of a Ct result below 27, it would be judicious to reanalyze each species of the multiplex in simplex to limit the risk of a hidden minor species. This should be defined according to the malaria epidemiology of each area and the probability of having mixed-infection. However, the limit of detection of the method was not impacted when the two species were far from the detection limit of the method. For thirteen samples, we also compared the capacity of real-time amplification or microscopy to detect mixed-infections. As expected, real-time PCR was more sensitive (Supplementary Table S3).

**Table 7:** Detection of *P. falciparum and P. vivax* in real mixed infection templates.

Artificial blood mixed-infection	Respective parasitaemia	Cycle threshold ± standard deviation		
		Duplex-PCR reagents		Simplex reagents
		*P. falciparum*	*P. vivax*	
*P. falciparum–P. vivax*	100 p/µl–100 p/µl	31.0 ± 0.3	31.5 ± 0.3	
*P. falciparum–P. vivax*	100 p/µl–10 p/µl	29.5 ± 0.4	33.0 ± 0.9	
*P. falciparum–P. vivax*	100 p/µl–1 p/µl	29.5 ± 0.5	–	
*P. falciparum–P. vivax*	10 p/µl–100 p/µl	32.0 ± 0.3	30.1 ± 0.2	
*P. falciparum–P. vivax*	10 p/µl–10 p/µl	32.3 ± 0.4	32.0 ± 0.1	
*P. falciparum- P. vivax*	10 p/µl–1 p/µl	32.0 ± 0.1	37.0 ± 0.3	
*P. falciparum–P. vivax*	1 p/µl–100 p/µl	41.5 ± 1.1	29.0 ± 0.3	
*P. falciparum- P. vivax*	1 p/µl–10 p/µl	35.0 ± 0.3	32.0 ± 1.5	
*P. falciparum–P. vivax*	1 p/µl–1 p/µl	34.5 ± 0.3	36.3 ± 0.6	
*P. falciparum*	100 p/µl	30.2 ± 0.5	–	30.0 ± 0.4
*P. falciparum*	10 p/µl	33.0 ± 0.6	–	33.0 ± 0.0
*P. falciparum*	1 p/µl	36.2 ± 0.6	–	36.4 ± 1.5
*P. vivax*	100 p/µl	–	30.2 ± 1.3	29.4 ± 0.1
*P. vivax*	10 p/µl	–	31.1 ± 0.2	31.0 ± 0.1
*P. vivax*	1 p/µl	–	36.0 ± 0.9	35.0 ± 0.5

### Robustness of the method

A depth analysis of the possible reasons of deviation of the results was carried out (freezing/thawing DNA, primers and probe degradation; temperature variation ate the hybridization step ± 1%; pipetting variation during mix preparation ± 10…). The acceptable limit was set at 10%.

A series of 10 freezes/thaws of *P. falciparum* primers and probes has been tested. A drift of 6.3% was observed below the acceptable limit of 10%.

**Figure 2:**
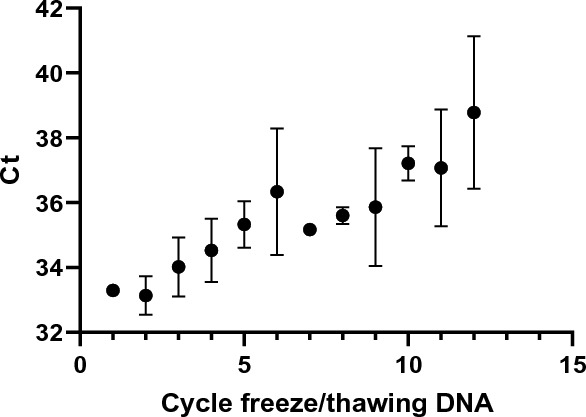
Impact of freezing/thawing of *Plasmodium falciparum* sample DNA in 30 µl-aliquots.

However, the number of freezing/thawing cycles had a major impact on DNA integrity of the samples, including the IQC. A drift of 15% was observed after 14 freezing/thawing cycles even when DNA was stored in aliquots of 30 µl (Fig. 2). Aliquoting IQC in small volume was therefore implemented to avoid several freezing/thawing cycles of each aliquot.

After choosing the best temperature for denaturation (95 °C) and amplification (60 °C), we determined the Maximum Permissible Error (MPE) of the StepOnePlus™ thermocycler in case of a temperature shift through time. The MPE is also required during metrological verification to establish the conformity of the thermocycler. The six parameters have been analyzed at several annealing temperature. By taking the plasmid as a template, we observe variable results with a reduction of Ct values for *P. falciparum, P. vivax* and *P. knowlesi* when the annealing temperature increased (Supplementary Fig. S2).

## Discussion

According to WHO, the microscopic examination of blood smears stained with Giemsa is the gold standard for diagnosis^1^. In a context of low transmission and elimination, the skills of microscopists are difficult to maintain and the asymptomatic carriers are difficult to detect. qPCR remains the most sensitive and species-specific methods compared to microscopy, ELISA or LAMP PCR^16^.

In this study, we implemented a robust, sensitive and rapid multiplex real-time PCR approach to detect the five human malaria species in venous blood collected on EDTA-vacutainer or capillary blood collected on filter paper in the context of the 15,189-accreditation requirements. The limit of detection (LoD) from DNA extracted on 200 µl of venous blood was 0.25 parasites/µl for *Plasmodium falciparum, P. vivax* and *P. knowlesi*, 1 parasite/µL for *P. ovale* and 5 parasites/µl for *P. malariae*. From 4-punches of dried blood spot, we achieve the limits of 5 parasites/µl for *Plasmodium vivax*, 20 parasites/µl for *P. falciparum*, *P. ovale* and *P. knowlesi* and 125 parasites/µl for *P. malariae.* For active detection campaigns which could include thousands of samples, we also implement a real time PCR targeting the genus *Plasmodium *spp. to facilitate the first screening for malaria. The detection limits determined for this method were similar to the one observed with the species-specific method for each species. This first round of detection of *Plasmodium spp.* carriers aims to limit the time and cost spending during active case detection campaign. The cost of this method has been evaluated at 20$/sample analyzed in triplicate for *Plasmodium *spp. This cost is competitive compared to the 15–40$/sample analyzed in single well of the different commercial kits or previously published methods^17^. The species-specific approach to detect the five human malaria species was around 27$/sample (prices including DNA extraction and qPCR without human resources). However, qPCR will always be an expensive method compare to RDTs but the only performant one to detect the last parasite carriers.

This method has been designed to reduce costs but also sample usage. As such we propose a duplex *P. falciparum*/*P. vivax* and a triplex *P. malariae*/*P. ovale*/*P. knowlesi*, in a final volume of 12 µl, containing 1 µl of DNA, tested in triplicate for each sample. Generally, previously published real time PCR used a final volume chosen of 25 µl containing two to five microliters of DNA^13,18,19^. During implementation, 5 µl of DNA has been tested to increase sensitivities but the impact on the detection limits was not high enough comparing to the consumption of the matrix (data not shown). However, we observed a clear impact on the detection limits when the number of targets within each multiplex was reduced. Four species within the same well as Shokoples et al. described, generated higher interferences and a drastic drop of the detection limits. Even using a duplex approach, we lost in sensitivity in case of mixed infection. In our transmission settings, French Guiana, the probability of mixed infection *P. falciparum*/*P. vivax* is low, 0.6% ± 0.3 over the past four years (data not shown). We therefore considered to analyze in simplex the second target when the first one presented a Ct value below 27. The other possibility to avoid to reanalyze samples, is to adapt the content of each multiplex. Each laboratory implementing this method would have to define their own diagnosis flowchart according to the epidemiological situation of malaria and the number of mixed-infections in their area. During our validation process, mixed infections with low parasitemia of *P. vivax* (around 1 parasites/µl or below) were not detectable if a *P. falciparum* infection of about 100 parasites/µl was also present. Generally, a 100-fold difference in concentration could impact the detect limit for diagnosis of the minor species.

This validated method also include the amplification of the human β2Megaglobuline gene to control DNA extraction. With a minimum Ct value to validate the results, this allow to confirm the volume of blood analyzed as well as the different steps of the DNA extraction. If the Ct values are not correct, the DNA extraction is repeated. However, in case of leukopenia or hyperleukocytosis, the Ct value will stay outside the range even if the volume of blood is correct. Those cases are rare and additional information such as complete blood count information are required. However, the result for malaria diagnosis is correct.

Malaria elimination is defined by the WHO as the interruption of local transmission of a specific species, in a defined geographical area, following activities specifically carried out for this purpose^1^. This multiplex real-time PCR method has been used during a mass screen and treat (MSAT) approach, Palustop, in French Guiana between October and December 2017 in the municipality of St Georges de l’Oyapock^15^. Using this real-time PCR, 1501 samples were analyzed from patients living in endemic area, 79% without symptoms and between 2 month and 93-year-old. The sex ratio was 0.88. One hundred positive samples have been identified, 90 with *Plasmodium vivax* and 10 with *Plasmodium falciparum*. No mixed-infection has been observed. The general prevalence of malaria using this method, has been determined at 6.6% [5.3–7.9], 74% of the people were asymptomatic. Among them, only nine samples out of 100 were positive by RDT^15^. These results show that RDT is not sensitive enough to detect asymptomatic carriers. In fact, in French Guiana, malaria has decreased for almost 15 years. Since 2005, *P. vivax* predominates but the proportion of *P. falciparum* on the Guiana Shield (region encompassing French Guiana, Guyana, Surinam, part of Brazil, Venezuela and Colombia) is one of the highest of South Americas. *P. malariae* transmission could sporadically occurred in the deep forest related to a wild fauna transmission with a very low prevalence. With a little more than one hundred cases in 2021, the territory targets malaria elimination for 2025. In this context, various operational research projects and active case detection have been initiated since 2017^15,20^.

Recently, ultra-sensitive qPCR (us-qPCR) have been developed to detect asymptomatic low-density malaria infections in endemic areas^21^. The term ultra-sensitive refers to the use of genes with higher copy number (i.e. *pfvarATS* for *P. falciparum*, or *pvcox1* for *P. vivax*) and/or increasing the volume of blood to be extracted. These PCR generally target *P. falciparum* and *P. vivax* carriers and aim to detect asymptomatic carriers with low parasitemia acting as parasite reservoir^22^. However, these methods achieve sensitivities below the threshold of one gametocyte/µl of blood which is considered as the limit to transmit malaria to mosquitoes^23^. When an individual is positive by qPCR, if his gametocytemia is below this threshold, he will not contribute to malaria transmission^24^. However, fluctuation of this parameter is poorly known and a longitudinal follow-up of asymptomatic carriers would be interesting in order to describe gametocytemia through time in asymptomatic carriers. This knowledge would help us to define, the benefit of implementing one diagnosis method or the other according to their sensitivity.

## Conclusion

We established a real time PCR method that can detect and identify the five human malaria parasites (*P. falciparum, P. vivax, P. malaria, P. ovale and P. knowlesi*) from whole venous sample and dried blood spot using a small quantity of DNA extract. Composition of each multiplex amplification should be adapted to the local epidemiological situation including the prevalence of mixed-infections.

This PCR requires equipment and well-trained personnel. The DNA extraction step is time consuming and treatments will probably have to be prescribed in the following days. Despite these limitations, this PCR is accredited in our lab according to the norm ISO 15,189 for medical biology which is strict. This quality process confirms the high performances, robustness and stability of the method. Furthermore, during active malaria detection campaigns the method has demonstrated its feasibility and usefulness in elimination settings.

## Materiel and methods

### Blood samples

Blood samples were obtained from blood venipuncture and collected either from patients consulting for fever in a health structure in French Guiana, France mainland or during active case detection campaigns. The parasitemia estimation was determined by microscopy and thin smear. We observed at least 50 fields of 250 red blood cells on average and counted the number of parasitized red blood cells using the cell counter. If no parasitized red cells are identified, we observed up to 100 fields then perform a thick film. If parasites are observed, we calculated the percentage of parasitized red blood cells or the parasitaemia following WHO recommendations. The parasitemia was determined by two readers and eventually a third one in case of discrepancies above 20% differences.

### DNA extraction

DNA was extracted from 200 µl of venous blood or four punches of capillary blood collected on filter paper using the QIAamp® genomic DNA kits (Qiagen) according to the manufacturer’s instructions. DNA elution volume was 100 µL in AE buffer when 200 µl of whole blood was extracted and 75 µl of AE buffer when 4-punches of diameter 3 mm of dried blood spots were extracted. The same protocol was used for all samples and external quality samples.

### Primers and probes

The primers and the probes (TaqMan) have been synthesized by Applied Biosystems and purified by high-performance liquid chromatography when labelled (Table 8). The β2-megaglobulin gene (β2MG) was used as an internal control of DNA extraction.

**Table 8:** Primers and probes used for identification of *Plasmodium* species targeting *18S* genes.

Species	Primer or probe	Sequence 5′–3′	Fluorescent label	Reference
Duplex				
* P. falciparum*	Fal-F	CCGACTAGGTGTTGGATGAAAGTGTTAA	VIC-MGBNFQ	Shokoples et al.^12,18^
	Fal-R	AACCCAAAGACTTTGATTTCTCATAA		
	Fal probe	AGCAATCTAAAAGTCACCTCGAAAGATGACT		
* P. vivax*	Viv-F	CCGACTAGGCTTTGGATGAAAGATTTTA	NED-MGBNFQ	
	Viv-R	AACCCAAAGACTTTGATTTCTCATAA		
	Viv probe	AGCAATCTAAGAATAAACTCCGAAGAGAAAATT		
Triplex				
* P. malariae*	Mal-F	CCGACTAGGTGTTGGATGATAGAGTAAA	FAM-MGBNFQ	Shokoples et al.^12,18^
	Mal-R	AACCCAAAGACTTTGATTTCTCATAA		
	Mal probe	CTATCTAAAAGAAACACTCAT		
* P. ovale*	Ova-F	CCGACTAGGTTTTGGATGAAAGATTTTT	VIC-MGBNFQ	
	Ova-R	AACCCAAAGACTTTGATTTCTCATAA		
	Ova probe	CGAAAGGAATTTTCTTATT		
* P. knowlesi*	Knw-F	CTAAAATGCGCACAAAGTCGAT	NED-MGBNFQ	De Canale et al.^13^
	Knw-R	GCAGTTAAAACGCTCGTAGTTGAA		
	Knw probe-1	CGGAGGCATCAGTTAT		
Simplex				
* Plasmodium* spp.	Spp-F	AGCTCTTTCTTGATTTCTTGG	VIC-MGBNFQ	Hassanpour et al.^14^
	Spp-R	CAGACAAATCATATTCACGAACT		
	Spp probe-1	AAACGGCCATGCATCACCAT		
Simplex				
Human β2-megaglobulin	2βMG-F	TGAGTATGCCTGCCGTGTGA	FAM-MGBNFQ	Shokoples et al.^12,18^
	2βMG-R	ACTCATACACAACTTTCAGCAGCTTAC		
	2βMG probe	CCATGTGACTTTGTCACAGCCCAAGATAGTT		

The targeted sequences of the *18 S rRNA* gene of *P. falciparum, P. vivax, P. malariae, P. ovale* and *P. knowlesi* (Accession number M19172.1, U93233.1, M54897.1, KF696363.1 and PKNH_0320900 respectively) were cloned into five different pEX-K4 plasmids (Eurogentec, Belgium) as Internal Quality Control (IQC) to continuously monitor the performances of the methods. IQC were used at a concentration of 1000 copies/µl in order to obtain a Ct around 30. This value has been chosen to observe potential drifts of the real time PCR from one PCR to another. IQC results were analyzed using the standardized WESTGARD rules used in medical biology. Serial dilutions of the plasmids were realized in nuclease free water from 10^12^ copies to 1 copy per µl in order to artificially constitute mixed-infections. Negative controls included TE buffer (10 mM Tris–HCl [pH 8], 1 mM EDTA).

### Real time PCR

The real-time PCR reaction consisted of 450 nM of each primer, 125 nM of probe, 5X TaqMan Universal Master Mix (Applied Biosystems, USA), and 1 μl of DNA in a 12 μl final volume. Reactions were performed on the StepOnePlus® (Applied Biosystems, USA). The following cycling conditions were applied: 50 °C for 2 min, 95 °C for 10 min, and 45 cycles of 95 °C for 15 s and 60 °C for 1 min. Fluorescence data was collected during the annealing/extension step at 60 °C.

Amplifications were performed in three separate reactions. The detection of *P. falciparum* and *P. vivax* in the first PCR and *P. malariae*, *P. ovale* and *P. knowlesi* in the second one was realized in triplicates to increase sensitivity and robustness of the method.

Cycle Threshold (Ct) values were analyzed by setting the threshold at 0.02 in order to harmonize the data from one experiment to another. The control of DNA extraction was realized using a simplex PCR which amplify the β2Megaglobulin Human gene. The manipulations were carried out in a single well and decision thresholds for venous blood was established at Ct ≤ 24 and for dried blood spot at Ct ≤ 33.

### Repeatability and reproducibility of the real time PCR

Repeatability (intra-assay variation) have been done on two samples per species (Table 9). For *P. falciparum* and *P. vivax* we used patient’s samples with parasitemia expressed with percentage. Due to a valuable and limited matrix, especially for *P. malariae, P. ovale* and *P. knowlesi* species, the number of repetitions was limited and External Quality Assessment (EQAs) with parasitemia expressed with parasites per microliters were used.

**Table 9:** Parasitemia of samples used to evaluate the repeatability of the method.

	*P. falciparum*	*P. vivax*	*P. malariae*	*P. ovale*	*P. knowlesi*
High-parasitemia	5.6%	2.2%	125 p/µl	200 p/µl	2000 p/µl
Low-parasitemia	0.005%	0.01%	20 p/µl	5 p/µl	1 p/µl

*p/µl* parasites/µl of blood.

The reproducibility (inter-assay variation) was determined using the internal quality control, including around 80 values obtained within a range of 7 to 15 consecutive days. All samples were analyzed in triplicates. Coefficients of variation (CVs) were then calculated based on the Mean of the CTs of the triplicate.

### Analytical sensitivities, specificities and detection of mixed infections

Sensitivity of the method to detect the five species by real-time PCR was evaluated by comparing the expected results with results obtained in EQAs. The specificities were first assayed in silico for the primers and probes by using BLAST software in order to avoid non-specific amplification. We prepared artificial mixed-infections (tenfold dilution factors) using blood of patients infected with *P. falciparum* and *P. vivax* or with plasmid containing the *P. falciparum* and *P. vivax* target sequences. These artificial mixed infections were analyzed in duplicates.

### Real-time PCR limits of detection

The analytical evaluation was carried out on samples for *P. falciparum* and *P. vivax* or EQA with known parasitemia for *P. malariae*, *P. ovale*, *P. knowlesi*.

For *P. falciparum* and *P. vivax*, blood samples including at least 90% of synchronous and in well-conditions rings were diluted in uninfected whole blood to achieve a concentration range of 1 × 10^3^ p/µl to 0.005 p/µl before extraction.

External quality controls were provided by WHO through UKNEQAS. The EQAs are received in two types of matrix: lyophilized whole blood and dried blood spot. The lyophilized whole blood was reconstituted in 500 µl of sterile water and extracted with QIAamp® genomic DNA kits (Qiagen). To create the same concentration ranges for *P. malariae*, *P. ovale* and *P. knowlesi*, DNA was directly diluted based on the initial parasitemia of the EQA after considering the concentration factor at the elution step of DNA extraction. Each sample or EQA was tested by real time PCR in 3 wells per parasitemia.

### Robustness and stability of reagents and DNA

The stability of the reagents has been evaluated using a succession of ten cycles of freezing / thawing for probe/primer set and fifteen cycles for *P. falciparum* DNA sample with a parasitemia of 2.1% and diluted to 1/10,000. A robustness test was also done to determine the Maximum Permissible Error (MPE) of the StepOnePlus™ machine. Several pairs of temperatures were tested for denaturation (94 and 95 °C) and hybridization/elongation (59 to 63 °C). The plasmids of the five spices was used and three negative venous blood sample was used to test the internal control β2-megaglobulin.

### Ethical consideration

For the present research, authors confirm that all experiments were performed in accordance with French regulations. The parasitology laboratory is receiving samples according to its mandate of National Reference Center since 1989 (Décret no 2016-806 du 16 juin 2016 relatif aux centres nationaux de référence pour la lutte contre les maladies transmissibles, for the 2023–2027 period). According to the French legislation (article L.1211-2 and related of the French Public Health Code), secondary use for scientific purpose of human clinical remaining samples collecting during care or surveillance are possible as long as the corresponding patients are informed and has not given any objection to them. To fulfill this requirement the Hospital brochure informed every patient through a brochure entitled ‘‘Information for patients” which detailed the opposition procedure. Solely the samples without immediate and delayed patient opposition, and/or from their legal guardians, have been included in this study.

Authorization associated to the samples collected during the active case detection campaign, Palustop, was approved by the *Comité de Protection des Personnes du Sud-Ouest et Outre-Mer 4* N° AM-36/1/CPP15-024 (French ethics committee). The database was anonymized and declared to the French Regulatory Commission (Commission Nationale Informatique et Libertés, CNIL, n°917186). Those samples collected by the National Reference Center were registered by the French Ministry for Research (declaration number DC-2010–1223; collection N°2). Solely the samples without immediate and delayed patient opposition, and/or from their legal guardians, have been included in this study.

## Supplementary Information

Supplementary Information.

## Data Availability

The authors declare that the data supporting the findings of this study are available within the paper and its supplementary information.
