# Supplementary material for: Molecular detection of human Plasmodium species using a multiplex real time PCR
Source: Sci Rep. 2023 Jul 14;13:11388. doi: 10.1038/s41598-023-38621-9 (PMC10349082; doi:10.1038/s41598-023-38621-9)
Supplement: Supplementary file 1 — Supplementary Information. [file 41598_2023_38621_MOESM1_ESM.docx]

# SUPPLEMENTARY MATERIAL

**Supplementary table S1: Limit of detection between nested and real time PCR for *Plasmodium spp,* *P. falciparum and P. vivax* collected on dried blood spot**

|  | **p/µl** | **100** | **50** | **20** | **10** | **5** | **1** | **0.5** |
| --- | --- | --- | --- | --- | --- | --- | --- | --- |
| *Plasmodium falciparum* | Nested PCR | + | + | + | - | - | - | - |
|  | Real Time PCR | 36.3± 0.5 | 36.6± 0.2 | 36.4± 0.5 | 37.6 ± NA | - | - | - |
|  | *Positive wells* | 2/2 | 3/3 | 2/3 | 1/3 | 0/3 | 0/3 | - |
| *Plasmodium spp.* | Real Time PCR | 33.0±0.3 | 33.4±0.5 | 35.1±0.8 | 36.0±0.5 | 33.0±0.7 | - | - |
|  | *Positive wells* | 3/3 | 3/3 | 6/10 | 4/10 | 3/10 | 0/3 | - |
| *Plasmodium vivax* | Nested PCR | + | + | - | - | - | - | - |
|  | Real Time PCR | 35.0± 0.5 | 36.0± 0.7 | 37.0± 0.7 | 38.3± 1.0 | 38.6± 1.3 | - | - |
|  | *Positive wells* | 3/3 | 3/3 | 3/3 | 3/3 | 3/3 | 0/3 | - |
| *Plasmodium spp.* | Real Time PCR | 32.0±0.4 | 32.0±0.5 | 35.0±0.5 | 35.0±1.1 | 36.3±NA | 37.0±NA | - |
|  | *Positive wells* | 5/5 | 5/5 | 9/10 | 9/10 | 1/5 | 1/5 | 0/3 |

Results are presented in mean Ct (cycle threshold) ± standard deviation according to the level of parasitemia (p/µl, parasite/µl). In green, the LoD chosen. NA: non-applicable. *Number of positive well in each real time PCR triplicate.

**Supplementary table S2: Detection of *P. falciparum and P. vivax* in duplex real-time amplification of mono-plasmid templates compare to mixed-plasmid template.**

| **Combination** | **Proportion** | **Ct*** ± SD* | |
| --- | --- | --- | --- |
|  |  | ***Duplex-PCR reagents*** | |
|  |  | ***P. falciparum*** | ***P. vivax*** |
| *P. falciparum- P. vivax* | 100p/µl - 100p/µl | 28.6±0.3 | 30.0±0.2 |
| *P. falciparum- P. vivax* | 100p/µl - 10p/µl | 29.0±0.2 | 37.0±2.6 |
| *P. falciparum- P. vivax* | 100p/µl - 1p/µl | 29.0±0.2 | - |
| *P. falciparum- P. vivax* | 10p/µl - 100p/µl | 33.0±0.2 | 30.2±0.8 |
| *P. falciparum- P. vivax* | 10p/µl - 10p/µl | 33.0±0.3 | 34.3±0.9 |
| *P. falciparum- P. vivax* | 10p/µl - 1p/µl | 33.2±0.6 | 36.2±0.3 |
| *P. falciparum- P. vivax* | 1p/µl - 100p/µl | 35.0±0.3 | 29.3±0.0 |
| *P. falciparum- P. vivax* | 1p/µl - 10p/µl | 35.0±0.1 | 34.1±0.3 |
| *P. falciparum- P. vivax* | 1p/µl - 1p/µl | 35.4±0.2 | 37.0±0.3 |
| *P. falciparum* | 100p/µl | 28.6±0.1 | - |
| *P. falciparum* | 10p/µl | 33.5±1.8 | - |
| *P. falciparum* | 1p/µl | 35.4±0.6 | - |
| *P. vivax* | 100p/µl | - | 28.6±0.2 |
| *P. vivax* | 10p/µl | - | 33.0±0.3 |
| *P. vivax* | 1p/µl | - | 37.0±0.6 |

*Ct for threshold cycle and SD for standard deviation

**Supplementary table S3: Detection of *P. falciparum/P. vivax* mixed infection in patients using microscopy, duplex or simplex real-time amplification.**

| **Patient** | **Microscopy**  (% of parasitemia) | | **Duplex amplification** | | | **Simplex amplification** | |
| --- | --- | --- | --- | --- | --- | --- | --- |
|  |  |  | Ct ± SD | | | | |
|  | *P. falciparum* | *P. vivax* | *P. falciparum* | *P. vivax* | *P. falciparum* | | *P. vivax* |
| Q219 | 0.02±0.0 | 0.32±0.0 | 26.3±0.4 | 24.0±0.1 | 27.0±0.2 | | 24.0±0.1 |
| Q221 | 0.01±0.0 | 0.21±0.0 | 23.0±0.3 | 23.0±0.3 | 23.0±0.4 | | 22.3±0.2 |
| R255 | - | 0.03±0.0 | 26.4±0.1 | 28.0±0.3 | 26.2±0.1 | | 25.2±2.2 |
| R262 | 0.24±0.0 | 0.63±0.2 | 22.0±0.2 | 21.4±0.2 | 22.0±0.5 | | 21.5±0.6 |
| R271 | **-** | 0.73±0.2 | **38.3±2.0** | 22.0±0.0 | **30.0±0.2** | | 22.0±0.1 |
| S008 | 0.11±0.1 | 0.15±0.2 | 22.6±0.1 | 26.5±0.2 | 22.3±0.1 | | 26.3±0.0 |
| S276 | 0.22±0.0 | **-** | 23.0±0.1 | **-** | 22.4±0.1 | | **37.0±0.4** |
| S620 | - | - | 32.0±0.4 | 29.3±0.4 | 33.4±0.4 | | 29.0±0.1 |
| S679 | 0.02±0.0 | 0.01±0.0 | 25.0±0.0 | 27.0±0.0 | 25.0±0.0 | | 27.0±0.0 |
| U485 | 1.23±1.04 | **1.32±NA** | 18.5±0.1 | **41.3±0.6** | 18.2±0.1 | | **27.2±0.1** |
| U516 | - | 0.01±NA | 27.0±0.0 | 28.0±0.1 | 26.5±0.2 | | 28.2±0.1 |
| U521 | 0.45±0.0 | **0.25±NA** | 20.4±0.1 | **-** | 20.2±0.1 | | **29.5±0.0** |
| U528 | - | 0.10±0.0 | 27.2±0.2 | 24.0±0.1 | 28.0±0.2 | | 23.5±0.1 |

**In bold**: discordant results. Explanation: a parasitemia putatively close to the detection limit for one species. In patient U485: species identification difficult because of an alteration of the parasite morphology. SD was calculated based on two readers for microscopy or triplicates for real-time amplification.

**Supplementary figure S1:** Comparison of P. vivax amplifications derived from tenfold dilution series between simplex and multiplex strategies. A. Simplex strategy. B. Duplex strategy. C. Ct differences observed of each of the dilution point representing according to the coefficient of variation of the method, 2.5%.

E: PCR efficiency, R²: Coefficient of determination, Y = ax + b: equation of the function.

***Pf***

***Pk***

***Pv***

***Pm***

***Po***

***β2MG***

**Supplementary figure S2: Determination of the Maximum Permissible Error (MPE) of the StepOnePlus™ depending on the denaturation and annealing temperatures.**
